# Lensless Tomographic Imaging of Near Surface Structures of Frozen Hydrated Malaria-Infected Human Erythrocytes by Coherent X-Ray Diffraction Microscopy

**DOI:** 10.1038/s41598-017-14586-4

**Published:** 2017-10-26

**Authors:** Viktoria Frank, Yuriy Chushkin, Benjamin Fröhlich, Wasim Abuillan, Harden Rieger, Alexandra S. Becker, Akihisa Yamamoto, Fernanda F. Rossetti, Stefan Kaufmann, Michael Lanzer, Federico Zontone, Motomu Tanaka

**Affiliations:** 1 Physical Chemistry of Biosystems, Institute of Physical Chemistry, University of Heidelberg, 69120 Heidelberg, Germany; 2 European Synchrotron Radiation Facility (ESRF), 38043 Grenoble, France; 3 Department of Infectious Diseases, Parasitology, University of Heidelberg, 69120 Heidelberg, Germany; 4 Institute for Integrated Cell-Material Sciences (WPI iCeMS), Kyoto University, 606-8501 Kyoto, Japan

## Abstract

Lensless, coherent X-ray diffraction microscopy has been drawing considerable attentions for tomographic imaging of whole human cells. In this study, we performed cryogenic coherent X-ray diffraction imaging of human erythrocytes with and without malaria infection. To shed light on structural features near the surface, “ghost cells” were prepared by the removal of cytoplasm. From two-dimensional images, we found that the surface of erythrocytes after 32 h of infection became much rougher compared to that of healthy, uninfected erythrocytes. The Gaussian roughness of an infected erythrocyte surface (69 nm) is about two times larger than that of an uninfected one (31 nm), reflecting the formation of protein knobs on infected erythrocyte surfaces. Three-dimensional tomography further enables to obtain images of the whole cells with no remarkable radiation damage, whose accuracy was estimated using phase retrieval transfer functions to be as good as 64 nm for uninfected and 80 nm for infected erythrocytes, respectively. Future improvements in phase retrieval algorithm, increase in degree of coherence, and higher flux in combination with complementary X-ray fluorescence are necessary to gain both structural and chemical details of mesoscopic architectures, such as cytoskeletons, membraneous structures, and protein complexes, in frozen hydrated human cells, especially under diseased states.

## Introduction

During the past decades, considerable efforts have been directed toward the development of label-free, high spatial resolution X-ray imaging methods. X-ray microscopy is particularly attractive for investigating biological specimens since it would not be limited by sample thickness (~0.5 µm), a problem regularly encountered by classical transmission electron microscopy^1^. Recently, soft X-ray microscopy has been applied to visualize internal structures of entire cells without the need of sectioning, staining, and subsequent three-dimensional reconstruction^2,3^. This enables one to obtain images with a spatial resolution of as good as 40 to 60 nm^4^. However, in spite of recent advances, these approaches are limited by the X-ray optics that is based on Fresnel zone plates. A typical challenge in X-ray transmission imaging is the poor lens efficiency (<22%) which can be overcome by the use of high doses. However, the radiation with a high dose often causes radiation damage to biological specimens, although there are several ways to circumvent this problem, e.g., by cryo-fixation and plung- freezing of the hydrated sample in liquid ethane^5^.

Recently, lensless, coherent X-ray diffraction imaging (CXDI) has been developed as an alternative method^6,7^. The approach is based on the phase contrast mechanism and shows a better dose efficiency compared to lens-based soft X-ray microscopy^5^. Here, an isolated sample, typically a few µm in size, is fully illuminated with a coherent beam (a plane wave illumination), and the intensity of diffracted beam is collected in the far-field regime. When the diffraction pattern is sampled finer than the Nyquist frequency, an iterative algorithm can be applied to retrieve the missing phases so that the real space image can be obtained by simple Fourier transformation^8,9^. This lensless imaging with a plane wave illumination allows imaging with a high spatial resolution, because the image quality is not deteriorated with the variation of sample positions (<500 nm). Following the first application of CXDI to amorphous samples^10^, this interesting technique has been used to visualize various biological specimens, ranging from dried E-coli^11^ and yeast^12^ to frozen hydrated *D*. *radiodurans* bacteria^13,14^, yeast cell^5^ and *Neospora caninum* ^15^. The application of tomographic coherent diffraction imaging was firstly demonstrated for dried human chromosomes^16^ and yeast cells^17^, but the experiments with dried samples suffered from substantial radiation damage. The reduction of radiation damage by cryo-fixation allowed for the extension of three-dimensional CXDI to microorganisms^15^. The implementation of ptychographic imaging further widened the scope of CXDI, now allowing objects larger than the beam size to be imaged^14,18^. Although ptychographic X-ray tomography is very sensitive to the sample position instabilities, recent development shows that this problem can be mitigated and spatial resolution ~18 nm can be achieved for biological specimens^19^.

In this study, we report the two- and three-dimensional coherent X-ray diffraction imaging of frozen hydrated human erythrocytes with and without infection by the human malaria parasite *Plasmodium falciparum*. *P*. *falciparum* is the most virulent form among the five *Plasmodium* species that can infect humans, being responsible for an estimated 429,000 deaths in 2015 alone^20^. The virulence of *P*. *falciparum* is attributed to the intra-erythrocytic developmental stages of the parasite and the ability of parasitized red blood cells to sequester in the deep vascular bed of inner organs, which, in turn, can lead to impaired tissue perfusion, hypoxia and inflammatory reactions^21^. Cytoadhesion is mediated by parasite-encoded immune-variant adhesins presented on the surface of the infected host cell in electron-dense protrusions, termed knobs^22^. Knobs elevate the adhesins above the surface and are critical for cytoadherence under flow^23^.

To date, there have been several attempts to visualize the internal structures of malaria-infected erythrocytes. Dubar *et al*.^24^ used a combination of phase imaging and X-ray fluorescence to localize hemozoin, a byproduct of hemoglobin degradation by the parasite, at sub-µm resolution in chemically fixed *P*. *falciparum*-infected erythrocytes before and after treatment with anti-malarial drugs. Jones *et al*.^25^ applied coherent diffraction tomography to visualize parasite compartments in chemically fixed, infected erythrocytes. Although a resolution of 70 nm was achieved, the endogenous topographic profiles of protein knobs could not be detected due to the irreversible denaturing caused by chemical fixation. In this study, we prepared so-called “ghost” cells (which are devoid of cytoplasm) by osmotic lysis and resealing, and captured the supramolecular architecture present on the cell surface by coherent X-ray diffraction imaging after cryo-fixation of the specimen in liquid ethane. Cryo-fixation enabled us to image the frozen hydrated cells close to their native state, while minimizing the radiation damage. For comparison, we also performed the coherent diffraction imaging of healthy, uninfected human erythrocyte ghosts, and discussed the spatial resolution for two-dimensional projection and three-dimensional tomography of uninfected and infected human erythrocytes.

## Methods

### Cell Culture

All of the experiments involving the use of human erythrocytes were approved by the Ethics Committee of the Medical Faculty, University of Heidelberg, Germany and the French Ministry of Research & Higher Education. The experiments were performed after obtaining informed consent from all voluntary donors in accordance with the relevant guidelines and regulations. *P*. *falciparum* clone FCR3^CSA^ was derived from the clonal line FCR3 (Gambia strain), possessing a capability to bind to chondroitin sulfate A (CSA)^26^. The selection of FCR3^CSA^ parasites with a high affinity toward CSA was performed by repeated selection (panning) of infected erythrocytes on CHO-K1 cells^27^. *P*. *falciparum* FCR3 strains were cultivated according to Trager *et al*.^28^ with A^+^ human erythrocytes supplemented with 10% human serum (Blood Bank IKTZ, Heidelberg), 10 mM hypoxanthine and 4 µg/ml gentamicin (Sigma, Neu-Ulm, Germany).

The cultures were synchronized^29^ and routinely selected by gelatin flotation to harvest knob-displaying parasites^30^. Parasitized erythrocytes (32 h post invasion) were enriched by using a magnetic column (Miltenyi Biotec, Auburn, USA) and achieved over 85% parasitaemias^31^.

### Sample Preparation

Erythocyte “ghosts” (cells after the removal of cytoplasm) were prepared from uninfected and *P-falciparum*-infected erythrocytes by osmotic lysis according to the modified preparation by Schwoch *et al*.^32^. First, a 40 ml portion of phosphate buffered saline (PBS) with 5 mM Na_2_HPO_4_, 150 mM NaCl and pH 7.4 was added to a 9 ml portion of blood. The suspension was centrifuged at 4000 g for 10 min at room temperature, and the supernatant was removed. This washing procedure was carried out three times, and a 40 ml portion of lysis buffer (5 mM Na_2_HPO_4_ without saline, pH 7.4) was added per 1 ml of sedimented erythrocytes to lyze the cells for 10 min at 0 °C. For resealing, NaCl was added to reach the final concentration of 2 mM NaCl, and the suspension was centrifuged at 4 °C for 30 min (15000 g). Finally, the fluffy pallets (erythrocyte ghosts) were washed three times with PBS. The quality of erythrocyte ghosts was carefully checked by immuno-fluorescence staining with the specific antibody to the extracellular domain of glycophorin C and the antibody specific to the cytoplasmic domain of band III (see Supplementary Fig. S1).

### Cryo-Fixation of Samples

**Figure 1:**
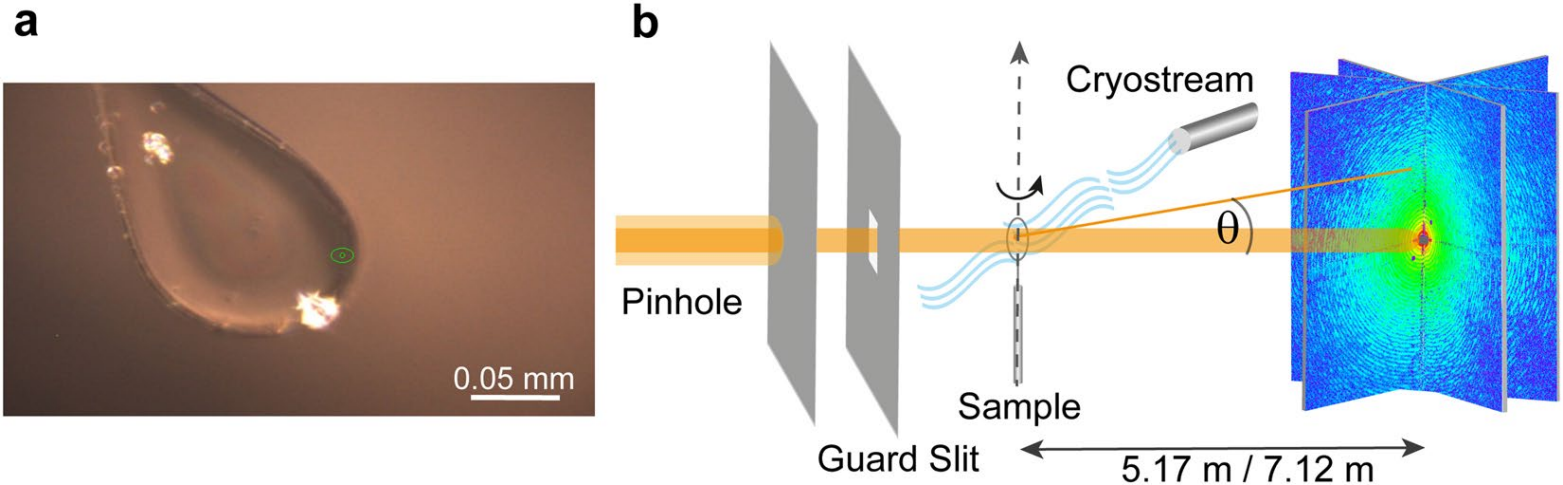
Experimental setup. (**a**) Microscopy image of the erythrocyte ghost suspension trapped in a cryo-loop and (**b**) schematic illustration of coherent X-ray diffraction imaging at ID10 beam line (ESRF). For three-dimensional tomography, the sample was rotated around the vertical axis.

The suspension of erythrocyte ghosts was trapped in a cryo-loop with a diameter ranging from 0.025 to 1.00 mm (Fig. 1a), and the excess water was dried in air for 30 s. To avoid the formation of crystalline ice that causes large background signals, 10 vol% of glycerol was added to the erythrocyte ghost suspension prior to plunging. Furthermore, to achieve a cooling rate faster than 10^6^ K/s, the cryo-loop (Hampton Research, USA) was plunged into liquid ethane. After the cryo-fixation the sample was placed on a sample holder under N_2_ cryo-stream to avoid the crystalline ice formation by condensation of water vapor (Fig. 1a).

### Coherent Diffraction Imaging

CXDI was performed at the ID10 beam line of the European Synchrotron Radiation Facility (ESRF, Grenoble, France). The cryo-fixed samples were mounted on the sample holder that possesses three translational and one rotational degrees of freedom. The sample was aligned in the beam using a mesh scan. During all the measurements the sample was kept under N_2_ cryo-stream at 100 K (Fig. 1b). The sample was illuminated with a coherent X-ray beam (10 µm × 10 µm, *E* = 7 keV), and the diffraction patterns were recorded with the two dimensional MAXIPIX 2 × 2 chip detector (516 × 516 pixels with a pixel size of 55 × 55 µm^33^.

For two-dimensional image reconstruction, the sample was illuminated for 300 s. The primary data processing, phase retrieval (see Method S2) and real space image reconstruction were performed with an in-house software. To get information on the 3D structure of the cells the samples were rotated in the horizontal plane covering −54 to +54° with a step-angle of 2° for uninfected cells and covering −60 to +60° with 1° steps for infected cells. The real space images were obtained from averaging 28 reconstructions using an iterative phase retrieval procedure^15^. The density distribution and total radiation dose were estimated following the procedure described in a previous account^15^. The experimental parameters for imaging are summarized in Table 1.

**Table 1:** Summary of experimental parameters for two-dimensional imaging and three-dimensional tomography.

	Healthy (Uninfected)		Infected	
	2D	3D	2D	3D
Intensity	9.78 × 10^9^	1.11 × 10^10^	9.78 × 10^9^	2.88 × 10^10^
	photons s^−1^	photons s^−1^	photons s^−1^	photons s^−1^
Sample-Detector Distance (L_SD_)	5.17 m	5.17 m	5.17 m	7.12 m
Number of Projections	1	55	1	121
Data matrix size	464 × 464	512 × 512 × 512	464 × 464	512 × 512 × 512
Exposure Time	300 s	150 s	300 s	20 s
Rotation Range	—	−54° to +54°	—	−60° to +60°
Total Radiation Dose	2.7 × 10^7^ Gy	8.55 × 10^8^ Gy	2.7 × 10^7^ Gy	6.48 × 10^8^ Gy

## Results

**Figure 2:**
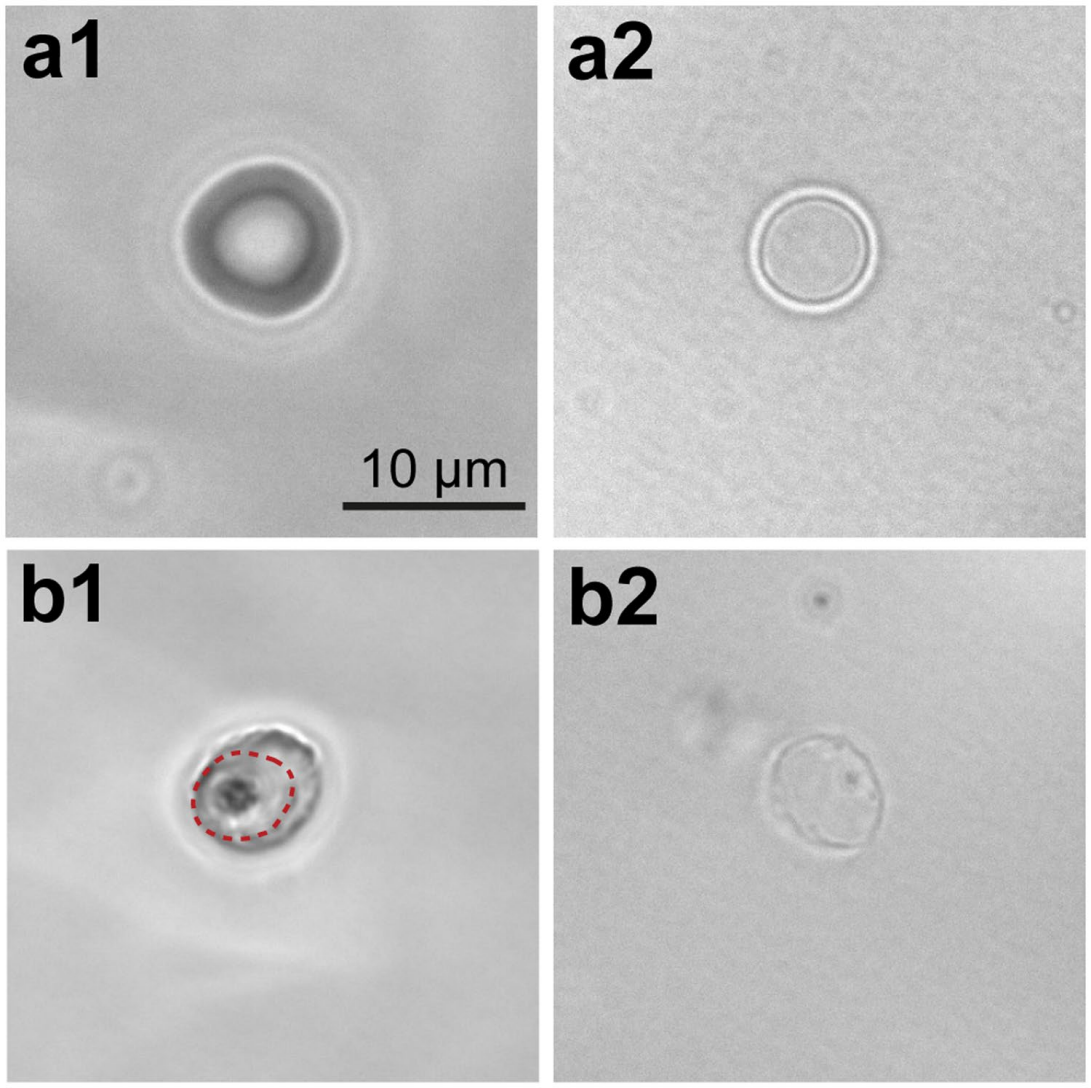
Optical phase contrast images of uninfected and infected erythrocytes and their ghosts. (**a**) Uninfected human erythrocytes before (a1) and after (a2) the removal of cytoplasm by osmotic lysis and resealing. (**b**) The corresponding images of a human erythrocyte infected by *P*. *falciparum* (t = 32 h). The parasite and vacuole membrane are visible in an intact erythrocyte (dashed line in b1) but lost after the osmotic lysis and resealing (b2).

Figure 2a depicts optical phase contrast images of a healthy, uninfected human erythrocyte before and after osmotic lysis. Prior to hypotonic lysis (Fig. 2a1), the erythrocyte had its natural discoidal shape with an apparent diameter of ~7 µm. After lysis and resealing, the erythrocyte became spherical with a diameter of ~4 µm (Fig. 2a2)^32,34^. As depicted in Fig. 2b, erythrocytes undergo significant morphological changes when they are infected with *P*. *falciparum*. 32 h post invasion, the outer surface of the parasitized erythrocyte became rougher (Fig. 2b1), and a parasite with its vacuole membrane was clearly visible. The rough, non-circular contour even after the removal of cytoplasm (Fig. 2b2) can be attributed to parasite proteins and membranous structures, such as knobs, that are inserted into the erythrocyte plasma membrane and anchored to the membrane skeleton of the host cell^35^. We confirmed the maintenance of the original membrane orientation by immuno-fluorescence staining with anti-glycophorin (extracellular domain) and anti-band III (cytoplasmic domain) antisera, as presented in Supplementary Fig. S1.

**Figure 3:**
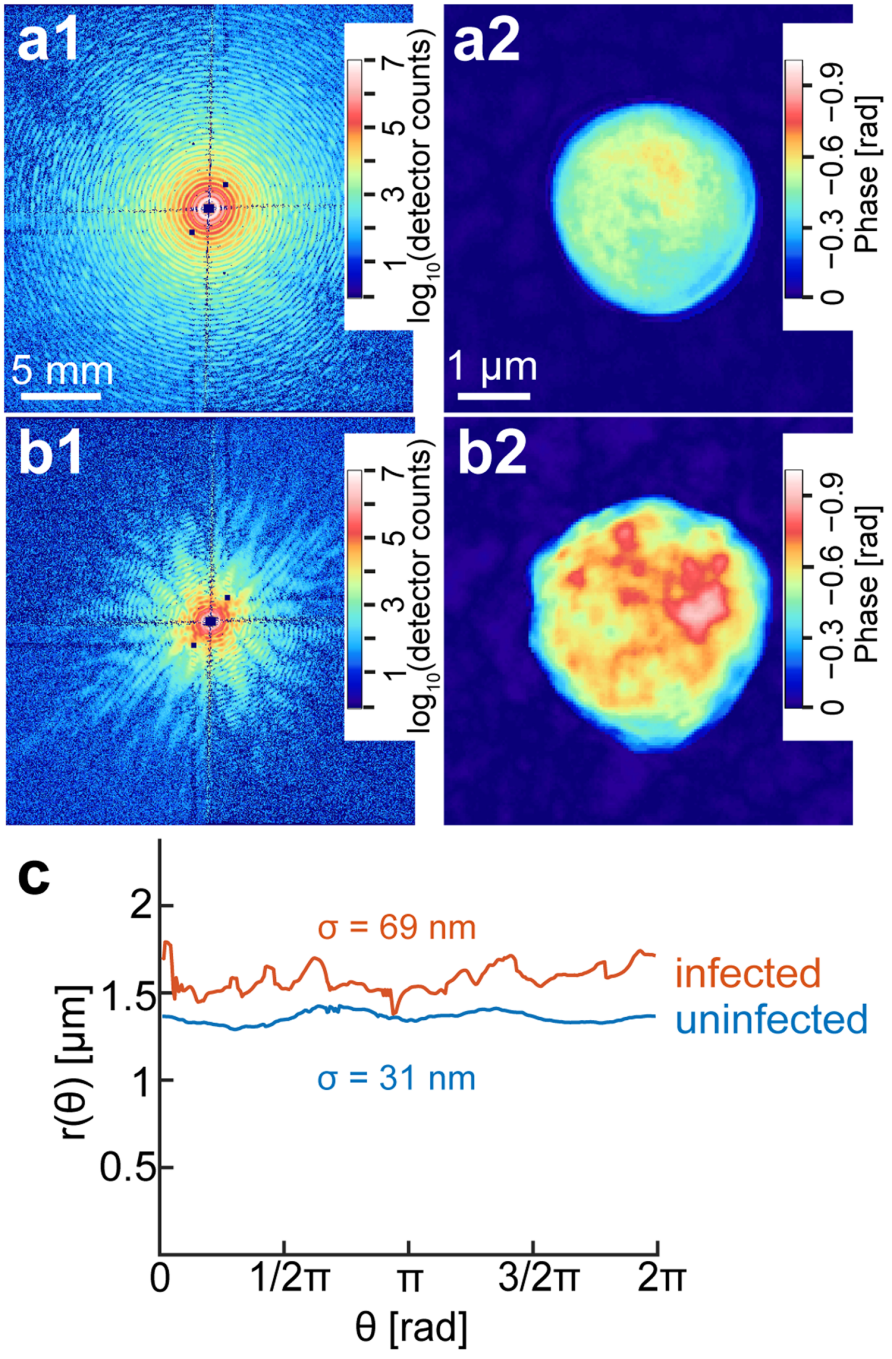
Two-dimensional, real space image reconstructions. (**a**1) Diffraction pattern measured by a two-dimensional detector and (**a**2) reconstructed real space image of a healthy, uninfected erythrocyte ghost kept in a cryo-loop. The corresponding data set of a human erythrocyte ghost infected by *P*. *falciparum* (t = 32 h) are presented in panels (**b**1) and (**b**2), respectively. (**c**) Radial distance from the center of mass to the periphery calculated from (**a**2 and **b**2).

Figure 3a1 represents the diffraction pattern from an uninfected erythrocyte ghost held in a cryo-loop. The recorded speckle pattern is concentric indicating a symmetric object in real space. Indeed, the real space image reconstructed from the speckle pattern (Fig. 3a2) indicates that the outer surface of a healthy, uninfected erythrocyte ghost is very smooth. Moreover, the electron density projected in two-dimensional space was more homogeneous, showing poorer characteristic features compared to that of an infected erythrocyte. In contrast, the diffraction pattern of an erythrocyte infected with *P*. *falciparum*, after the hypotonic lysis and resealing, exhibited distinct features in addition to the concentric speckles pattern (Fig. 3b1). The reconstructed real space image of the infected ghost (Fig. 3b2) shows that the cell surface became much rougher compared to that of an uninfected erythrocyte, which can be attributed to the formation of “knobs” on the cell surface. The structural features possessing a distinctly higher and more heterogeneous electron density can be attributed to the remaining parasite’s membranous structures inside the cell. Previous studies have shown that Maurer’s clefts and, possibly, fragments of the parasitophorous vacuolar membrane (which separates the parasite from the erythrocyte cytoplasm) remain attached to erythrocyte ghosts and, thus might contribute to the heterogeneous electron density^35^. Maurer’s clefts are membrane profiles of parasite origin within the cytoplasm of the host cell. They serve as an intermediary secretory compartment for parasite-encoded proteins en route to the host cell surface^36^.

Figure 3c represents the radial distance from the center of mass to the periphery plotted in polar coordinates for uninfected (blue) and infected (red) erythrocytes. The Gaussian roughness from the contour of an uninfected erythrocyte is σ = 31 nm, while an infected erythrocyte exhibits a distinctly larger roughness, σ = 69 nm. Such an increase in the surface roughness might be attributed to the increase in the surface density of protein knobs according to the development of parasites^37^. Moreover, it has been reported that the protein knob density is highly heterogeneous between different parasite strains^37^. For *P*. *falciparum* FCR3 strain used in this study, the knob densities on erythrocytes after ~30 h of infection have been measured by atomic force microscopy in a dry state, showing a large deviation from 2–4^37^ to 5–15 knobs per 1 µm^2^ ^38^. As the surface topography images for *P*. *falciparum* FCR3 strain are not available in the literature, we compared our results with the data calculated from the previously presented scanning electron microscopy (SEM) image of a dried erythrocyte in the comparable developmental stage of a parasite, σ = 65 nm (see Supplementary Fig. S3)^39^. CXDI and SEM images are both projected to two-dimensional planes, and the Gaussian roughness was calculated from the contour of each image. The results suggest that the topographic features of an infected erythrocyte captured by CXDI can be attributed to the protein knobs expressed on the surface of infected erythrocytes. However, the comparison of the roughness calculated from the projected CXDI image of a frozen-hydrated cell to the one calculated from an SEM image of a dried cell should be taken with a caution. Although the imaging of near surface topography of cells without denaturing still remains as a technical challenge, the obtained results show a potential of CXDI for imaging human cells under frozen-hydrated conditions.

The experimental spatial resolution for the two-dimensional reconstruction was estimated, following previous accounts^5,15^. For this purpose, we analyzed intensity line profiles of the ghost cells. Some well visible features with a distinct difference in phase shift of ~0.02 [rad], were used to estimate the spatial resolution of the reconstructed images. Line scans exhibited abrupt changes in the phase shift over 2 detector pixels, yielding the lateral resolution of Δ*x* ≥ 72 nm (see Supplementary Figs S4 and S5).

**Figure 4:**
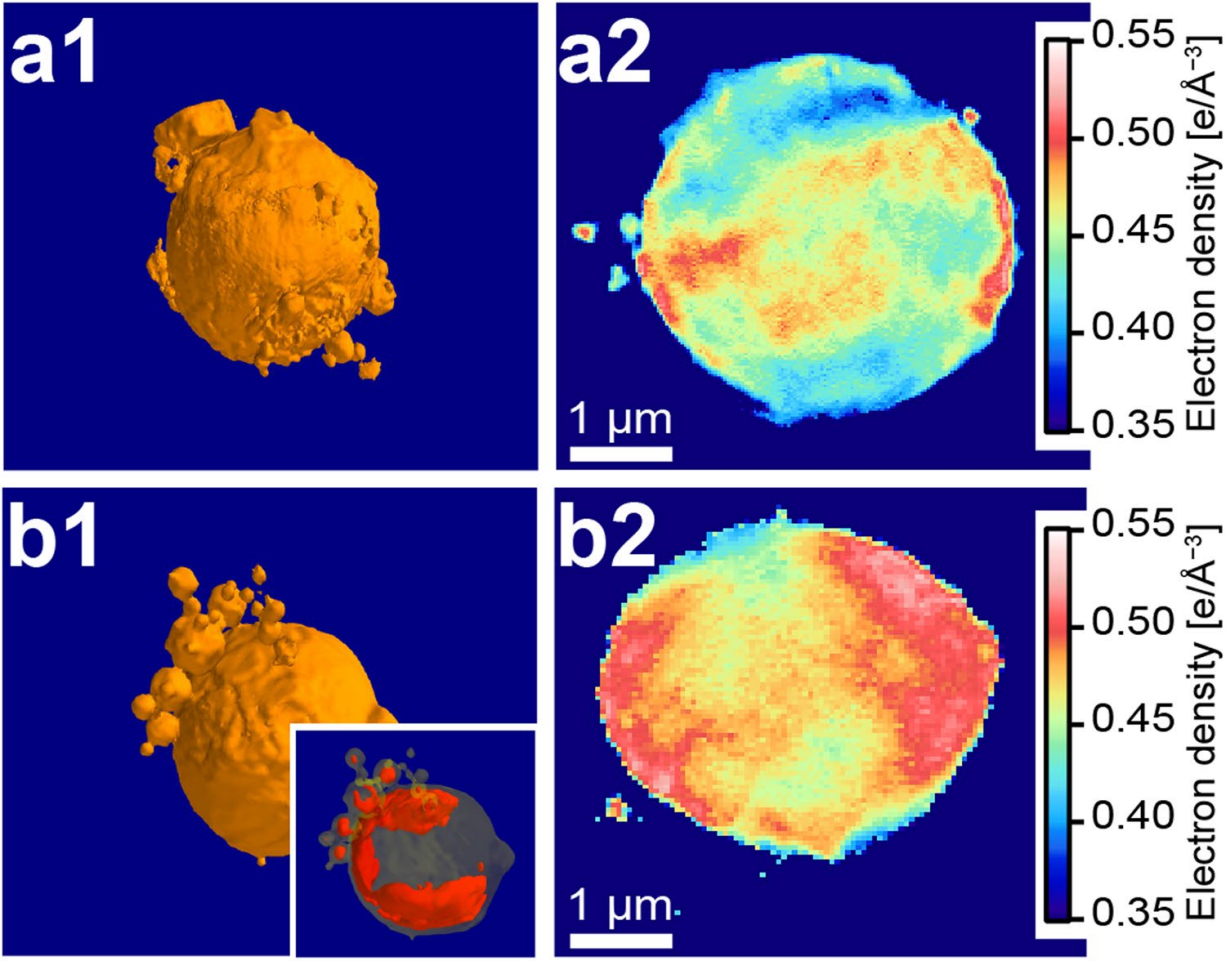
Real space image reconstruction from three-dimensional tomography (**a**1) Three-dimensional, iso-surface rendering of an uninfected human erythrocyte ghost, and (**a**2) electron density map of one selected slice. The corresponding data for malaria-infected erythrocyte (t = 32 h) are presented in panels b1 and b2, respectively. The red contour marks the electron-dense region in the inset of panel b1.

Since no radiation damage to the frozen hydrated erythrocyte ghosts could be detected, we further performed the three-dimensional, whole-cell tomography. The resulting three-dimensional iso-surface rendering of uninfected and infected erythrocyte ghosts is presented in Fig. 4a1 and b1, respectively. Both uninfected and infected erythrocyte ghosts exhibited some foreign objects on their cell membrane surfaces; most of them were spherical, while only a few had facets. The origin of the spherical objects can be attributed to vesicles formed during ghost preparation^34^, while the object with facets might coincide with salt crystals from the buffer. It should be noted that it is non-trivial to define the cell surface in the presence of such foreign objects. Since the manual removal of foreign objects often creates artifacts, we refrained from the calculation of surface roughness from the 3D tomograms.

Unlike projected two-dimensional images, three-dimensional tomograms allow features from the internal structure of the cells to be captured. Figure 4a2 and b2 show selected slices through the equatorial plane of uninfected and infected ghosts, respectively. The slice from an uninfected erythrocyte ghost (Fig. 4a2) suggests the presence of objects with a higher electron density near the membrane (*ρ* ~ 0.5 e/Å^3^), which can be attributed to the remaining spectrin cytoskeleton^34^. The electron density inside the uninfected erythrocyte ghost is mostly smaller than 0.5 e/Å^3^ and large fraction of internal volume reaches nearly the value of water (i.e. ice) *ρ* = 0.34 e/Å^3^. The remaining difference in electron density in the middle of the cell can be attributed to the remaining hemoglobin after the ghost preparation^32^. The electron density inside an infected erythrocyte ghost (Fig. 4b2) seems more heterogeneous (*ρ* ~ 0.45–0.55 e/Å^3^) compared to that in the uninfected ghost erythrocyte (Fig. 4a2), and the observed differences in the electron densities are related to the cell characteristics and cannot be explained by the limited scanning range (±54° against ±60°). The electron density inside an infected erythrocyte ghost is well above the electron density of water, suggesting the presence of internal structures with a higher electron density (Fig. 4b1, inset). Although it was not possible to capture characteristic structural features in detail, they might be attributed to the remaining membranous structures formed by *P*. *falciparum*, such as Maurer’s clefts^40^. As shown in Fig. 3a1 and b1, the signal intensity from the central speckles, which is necessary to calculate the absolute electron density, is blocked by the beam stop but this information can be recovered by the phase retrieval algorithm and used for density estimation.

**Figure 5:**
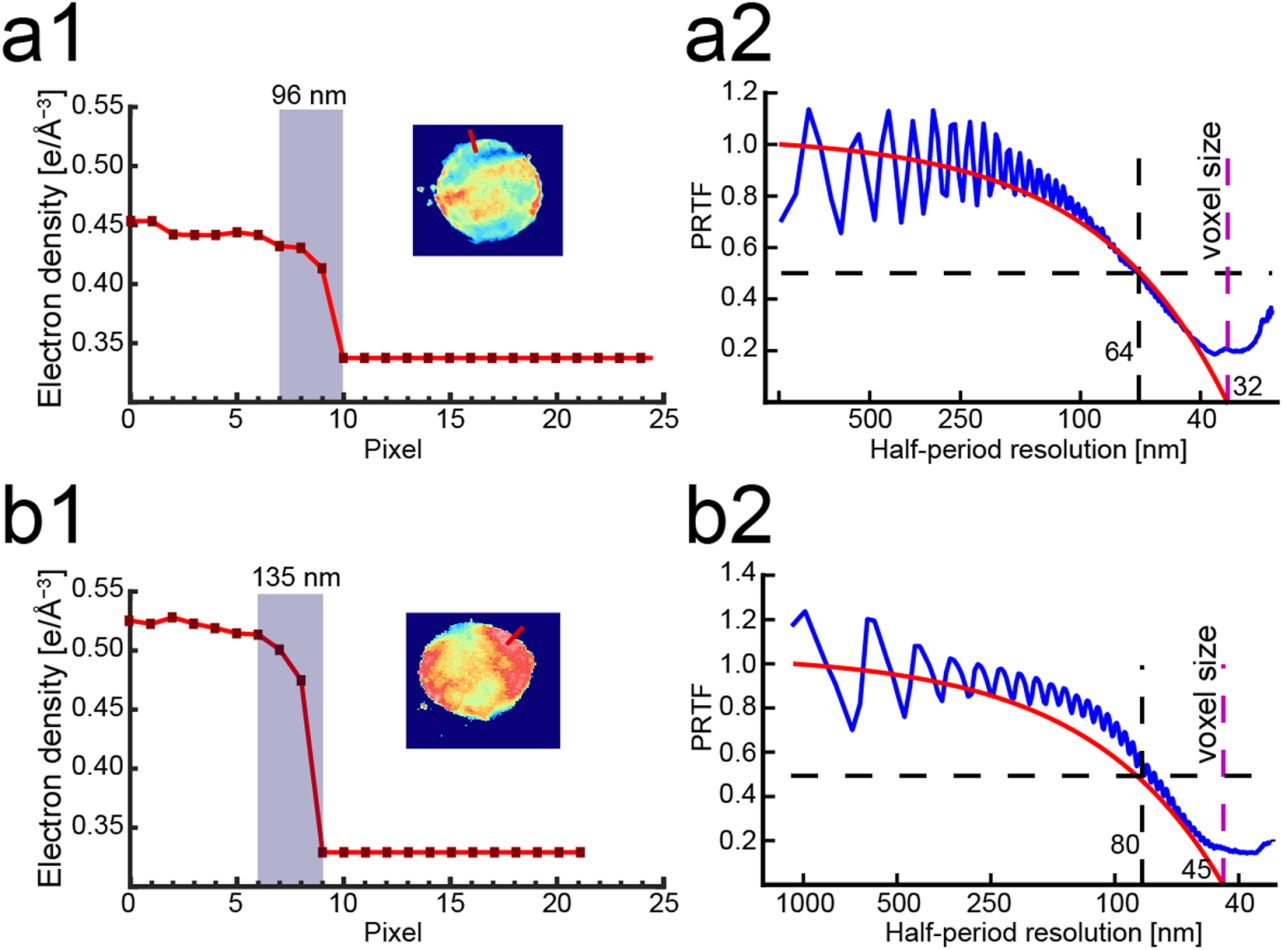
Estimation of spatial resolution in three-dimensional reconstruction. (**a**1) The line profile extracted from a slice of an uninfected erythrocyte (red line in inset) yields the lateral resolution of Δ*x*_pixel(uninfected)_ = 96 nm (gray boxes). (**a**2) Experimental phase retrieval transfer function (PRTF, blue) and theoretical modulation transfer function (MFT, red) plotted as a function of spatial frequency. From the cut-off threshold at PRTF = 0.5, one can gain the half-period resolution of Δ*x*_half(uninfected)_ = 64 nm. The corresponding data for malaria-infected erythrocyte (t = 32 h) are presented in panels b1 and b2, yielding the lateral resolutions of Δ*x*_pixel(infected)_ = 135 nm and Δ*x*_half(infected)_ = 80 nm.

Following the same procedure as was used for two-dimensional projections (see Supplementary Fig. S2), the spatial resolution of three-dimensional tomography was assessed from the electron density line profiles, yielding an apparent resolution of Δ*x*_pixel(uninfected)_ = 96 nm for uninfected erythrocyte ghosts (Fig. 5a1). As an alternative and a more quantitative measure of the resolution, we calculated the ratio between Fourier amplitude of the reconstructed image *A*(*f*) and square root of the experimentally measured intensity *Int*(*f*) as a function of frequency *f*, called phase retrieval transfer function (PRTF)^41,42^:

1 $$PRTF(f)=\frac{{\sum }_{f=const}A(f)}{{\sum }_{f=const}\sqrt{Int(f)}}.$$

PRTF = 1 coincides with the perfect matching, and the effective resolution is defined at the threshold PRTF = 0.5. Figure 5a2 represents the PRTF of an uninfected erythrocyte. As presented in Fig. 5, PRTF of an uninfected erythrocyte (blue) decreases according to the increase in spatial frequency, crossing 0.5 at around half-period resolution of Δ*x*_half(uninfected)_ = 64 nm. For comparative reasons, the theoretical modulation transfer function (MTF, red) is plotted in the same graph. Here the MTF is defined as:

2 $${MTF}(f)=\frac{\lambda {L}_{{SD}}}{{l}_{{pix}}{\rm{\Delta }}{x}_{{half}}}$$

where *λ* is the wavelength, *L*_SD_ the sample-detector distance, *l*_pix_ the pixel size, and ∆*x*_half_ the half-period resolution. Figure 5b1 and b2 show the line scan analyses and the PRTF and MTF of an infected erythrocyte, yielding the slightly poorer lateral resolutions of Δ*x*_pixel(infected)_ = 135 nm and Δ*x*_half(infected)_ = 80 nm. The corresponding data set from two-dimensional projections is presented in Supplementary Fig. S6.

Note that there is a difference between the resolution estimated from the line scan analysis and that estimated from the PRTF analysis. As the line scan is performed at a certain position in the image, e.g. the interface between cell and ice matrix, it is a local measure of the resolution. In contrast, the PRTF is calculated over the entire cell volume in 3D. However, the resolution of the reconstructed 3D image from the PRTF is anisotropic due to the limited angular range covered^39^. The resolution is higher in the direction perpendicular to the incoming beam and lower in the direction parallel to the beam. In our analysis, the PRTF yields the angular-averaged (isotropic) resolution of the entire image and thus it can deviate from the resolution obtained by the line scan analysis.

## Discussion

It is noteworthy that the resolutions we obtained for the frozen hydrated erythrocytes in this study are comparable to the ones reported for chemically fixed erythrocytes^25^, and the use of coherent diffraction imaging of frozen hydrated cells offers a unique advantage to capture the distinct roughening of the cell surfaces and the confinement of high electron density regions in the proximity of the erythrocyte plasma membrane. Although the spatial resolution needs further improvement, our data suggest that the lensless, coherent X-ray diffraction imaging of the whole cell under frozen hydrated conditions is a promising strategy for the label-free visualization of supramolecular architectures on and immediately below the plasma membrane, such as knobs and Maurer’s clefts, as demonstrated for *P*. *falciparum*-infected erythrocytes. Lensless, coherent X-ray diffraction imaging might further resolve parasite-induced changes in host cell membrane skeleton and how certain structural haemoglobin abnormalities interfere with this process^43^. Further combination with X-ray fluorescence microscopy would offer unique advantages toward the structural and chemical imaging inside biological cells^19,24^, which can be considered as another class of correlative imaging platforms based on X-ray.

## Electronic supplementary material

Supplementary Information
