# Supplementary material for: Lensless Tomographic Imaging of Near Surface Structures of Frozen Hydrated Malaria-Infected Human Erythrocytes by Coherent X-Ray Diffraction Microscopy
Source: Sci Rep. 2017 Oct 26;7:14081. doi: 10.1038/s41598-017-14586-4 (PMC5658481; doi:10.1038/s41598-017-14586-4)
Supplement: Supplementary file 1 — Supplementary Information [file 41598_2017_14586_MOESM1_ESM.pdf]

Supplementary information

Lensless Tomographic Imaging of Near Surface Structures of Frozen Hydrated Malaria-Infected Human Erythrocytes by Coherent X-Ray Diffraction Microscopy

Viktoria Frank[1,+], Yuriy Chushkin[2,*,+], Benjamin Fröhlich[1,+], Wasim Abuillan[1], Harden Rieger[1,3], Alexandra S. Becker[1], Akihisa Yamamoto[1,4], Fernanda F. Rossetti[1,4], Stefan Kaufmann[1], Michael Lanzer[3], Federico Zontone[2], Motomu Tanaka[1,4,*]

[1]Physical Chemistry of Biosystems, Institute of Physical Chemistry, University of Heidelberg, 69120 Heidelberg, Germany

[2]European Synchrotron Radiation Facility (ESRF), 38043 Grenoble, France

[3]Department of Infectious Diseases, Parasitology, University of Heidelberg, 69120 Heidelberg, Germany

[4]Institute for Integrated Cell-Material Sciences (WPI iCeMS), Kyoto University, 606-8501 Kyoto, Japan

[*]corresponding authors: chushkin@esrf.fr, tanaka@uni-heidelberg.de

[+] equal contributors

Supplementary Figure S1

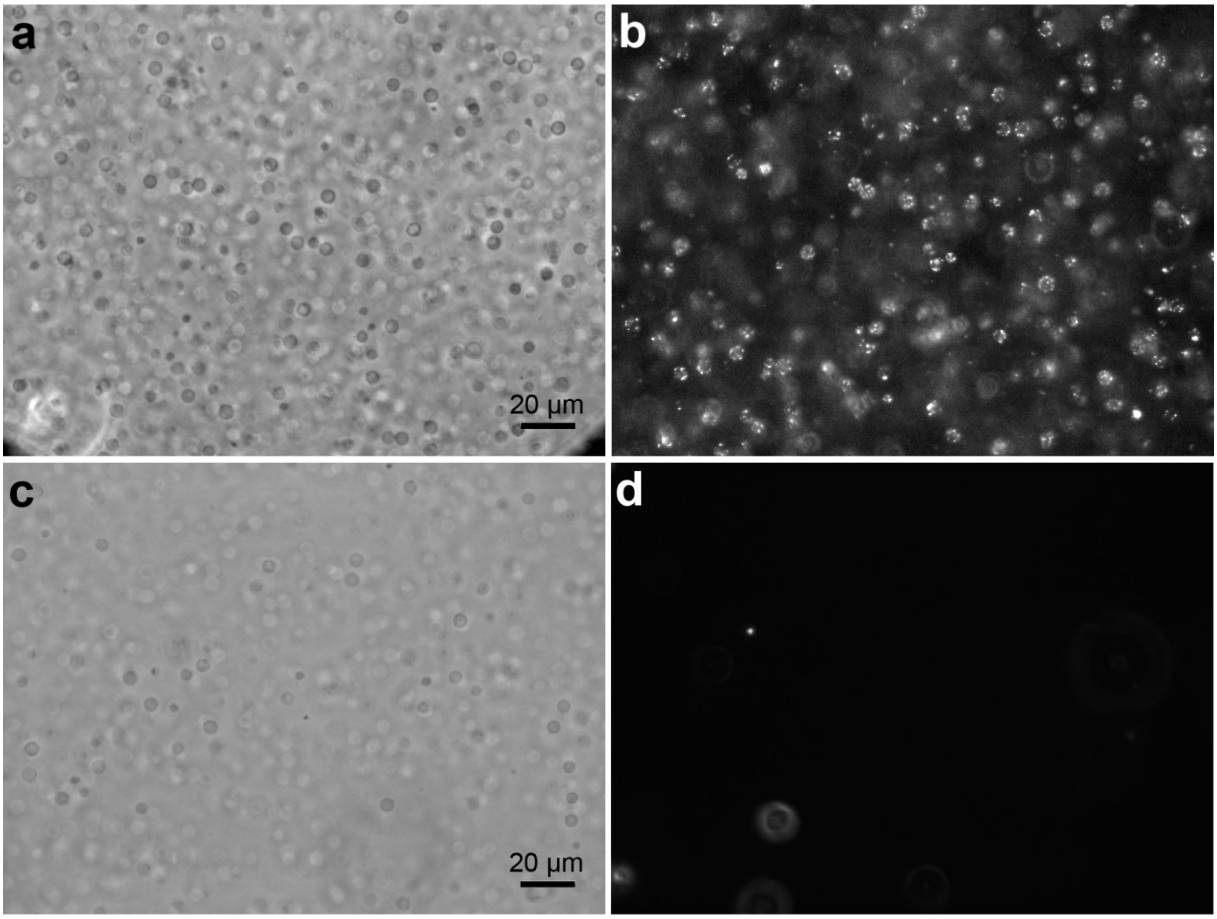

Figure S1. Maintenance of the membrane orientation in ghost cells. (a) Bright field image and (b) immuno-fluorescence (anti-glycophorin, extracellular domain) image of erythrocyte ghosts after osmotic lysis and resealing. (c) Bright field image and (d) immuno-fluorescence (anti-band III, cytoplasmic domain) image of erythrocyte ghosts after osmotic lysis and resealing.

Supplementary Method S2. Phasing Approach

2D images were obtained using a phase retrieval algorithm consisting of 4200 iterations of the hybrid-input-output algorithm (HIO) followed by 1800 iterations of the error reduction algorithm (ER) [1]. The support was refined using shrinkwrap method[2] only during the first 4200 iterations. The algorithm was applied to the 2D diffraction data cropped to a size of 464×464 pixels. The 20 best reconstructions obtained from random seeds were averaged to get the final 2D real space image of the specimen.

For the 3D reconstruction the 2D diffraction patterns were assembled into 3D diffraction volume using linear interpolation. The phase retrieval was applied on the 3D diffraction volume using 700 iterations of the HIO with the support refinement followed by 300 iterations of the ER. The 28 best reconstructions were averaged to get the 3D images of the specimens. Additional detail on the reconstruction procedure can be found in Chushkin et al 2014[3].

Supplementary Figure S3

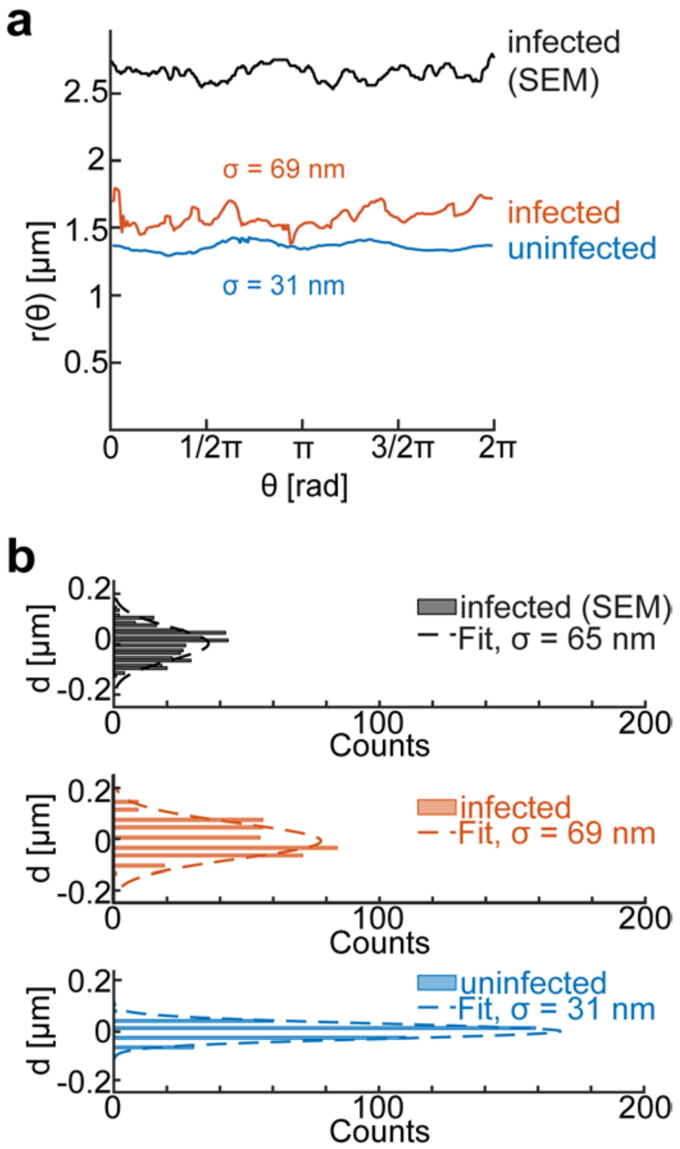

Figure S3. Surface profiles of healthy and infected erythrocytes from two-dimensional reconstructions. (a) Profiles of the radial distances from the center of mass to the cell rim r(θ) ($\Delta_\theta = 2\pi/360$) calculated from the 2D projections shown in Fig3 a2 and b2. Reference data (infected (SEM)) derived from scanning electron micrographs of infected erythrocytes from Fairhurst et al. 2012, Figure 2.[4] (b) Corresponding histograms of the fluctuation amplitude d = r(θ) - r_{mean}, fitted to a Gaussian function to determine the roughness parameter σ.

Supplementary Figure S4

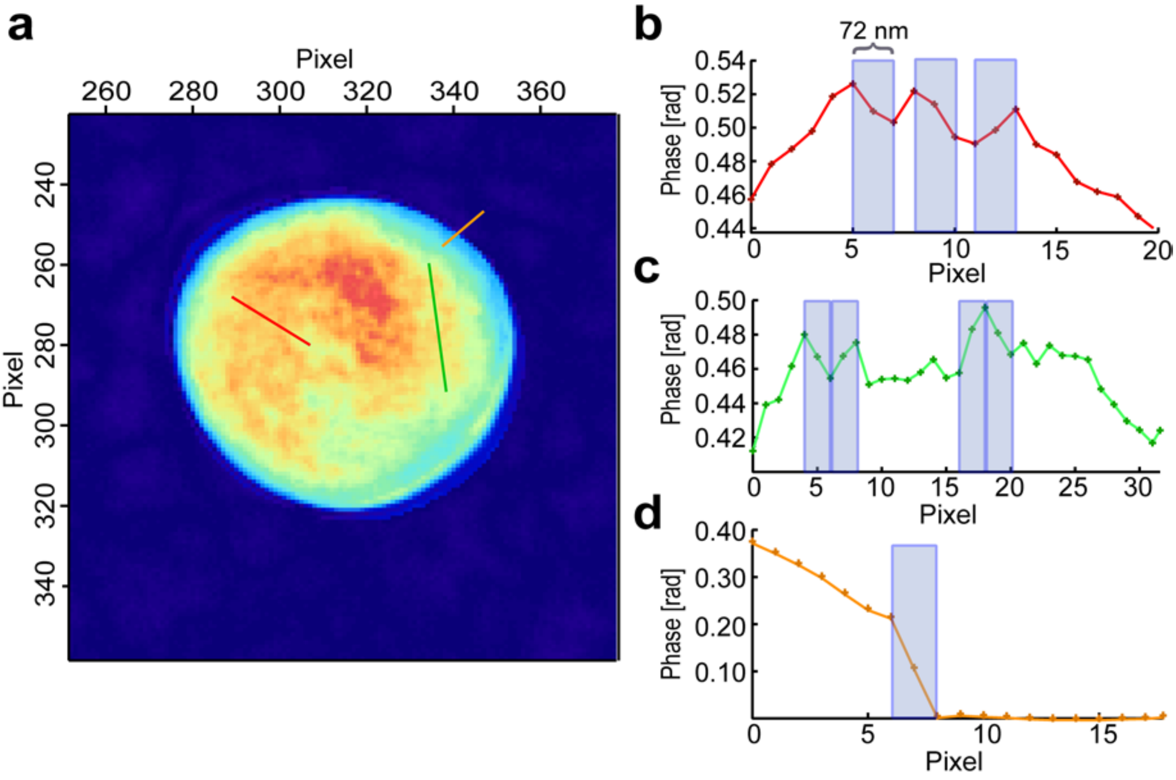

Figure S4. The spatial resolution of the two-dimensional projection obtained from the line profile analysis. (a) The analysis of the projected two-dimensional electron density map of a healthy erythrocyte ghost, corresponding to Fig.3a2 in the main text. (b, c, d) Three intensity line profiles extracted from the image. (1 pixel = 36 nm)

Supplementary Figure S5

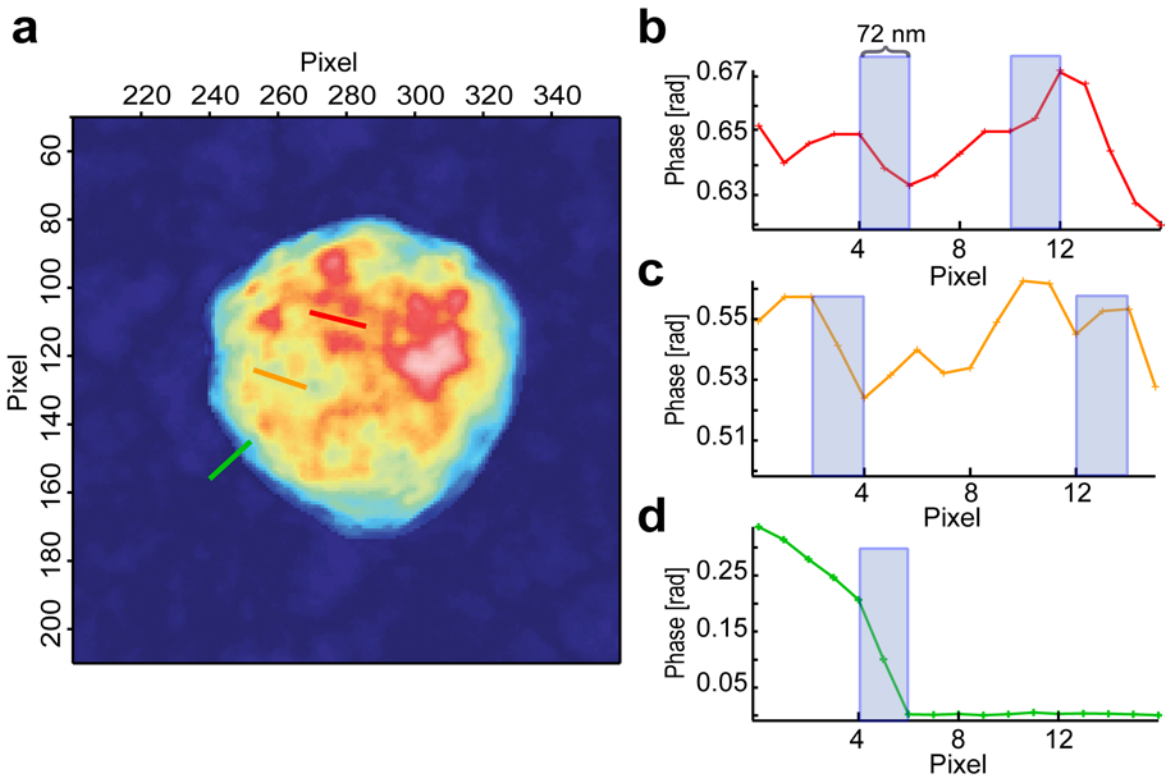

Figure S5. The spatial resolution of two-dimensional projection obtained from the line profile analysis. (a) The analysis of the projected two-dimensional electron density map of an infected erythrocyte ghost, corresponding to Fig.3b2 in the main text. (b, c, d) Three intensity line profiles extracted from the image. (1 pixel = 36 nm)

<u>**Supplementary Figure S6**</u>

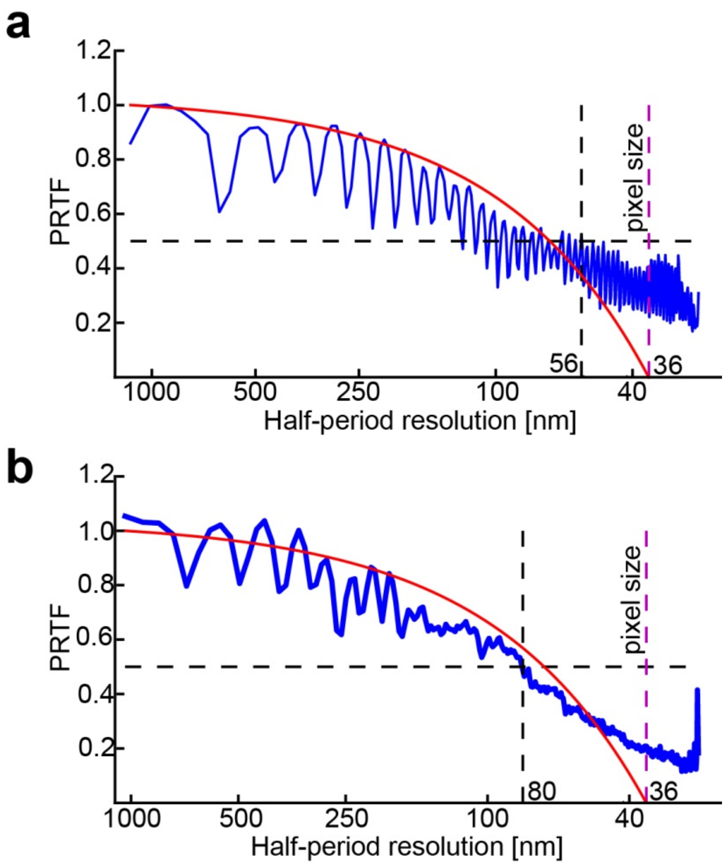

Figure S6. Phase retrieval transfer function (PRTF) from two-dimensional projections. (a) PRTF of a healthy erythrocyte yields $\Delta x_{half(healthy)}$ = 56 nm, while (b) PRTF of a malaria-infected erythrocyte yields $\Delta x_{half(infected)}$ = 80 nm.

1 Fienup, J. R. Phase retrieval algorithms: a comparison. *Appl. Opt.* **21**, 2758-2769 (1982).
2 Marchesini, S. *et al.* X-ray image reconstruction from a diffraction pattern alone. *Physical Review B* **68**, 140101 (2003).
3 Chushkin, Y. *et al.* Three-dimensional coherent diffractive imaging on non-periodic specimens at the ESRF beamline ID10. *Journal of synchrotron radiation* **21**, 594-599 (2014).
4 Fairhurst, R. M., Bess, C. D. & Krause, M. A. Abnormal PfEMP1/knob display on Plasmodium falciparum-infected erythrocytes containing hemoglobin variants: fresh insights into malaria pathogenesis and protection. *Microbes Infect.* **14**, 851-862 (2012).
